# Influence of Low-Temperature Cap Layer Thickness on Luminescence Characteristics of Green InGaN/GaN Quantum Wells

**DOI:** 10.3390/ma16041558

**Published:** 2023-02-13

**Authors:** Haoran Sun, Yuhui Chen, Yuhao Ben, Hongping Zhang, Yujie Zhao, Zhihao Jin, Guoqi Li, Mei Zhou

**Affiliations:** 1 Department of Applied Physics, China Agricultural University, Beijing 100083, China; 2 State Key Laboratory on Integrated Optoelectronics, Institute of Semiconductors, Chinese Academy of Sciences, Beijing 100083, China; 3 College of Science, China Agricultural University, Beijing 100083, China; 4 Science and Technology on Reliability and Environment Engineering Laboratory, School of Reliability and Systems Engineering, Beihang University, Beijing 100191, China

**Keywords:** InGaN/GaN MQW, cap layer, localized state, luminescence characteristics

## Abstract

GaN cap layer with different thicknesses was grown on each InGaN well layer during MOCVD growth for InGaN/GaN multiple quantum well (MQW) samples to study the influence of the cap layer on the photoluminescence (PL) characteristics of MQWs. Through the temperature-dependent (TD) PL spectra, it was found that when the cap layer was too thick, the localized states of the quantum wells were relatively non-uniform. The thicker the well layer, the worse the uniformity of the localized states. Furthermore, through micro-area fluorescence imaging tests, it was found that when the cap layer was too thick, the luminescence quality of the quantum well was worse. In summary, the uniformity of the localized states in the quantum wells and the luminescence characteristics of the quantum wells could be improved when a relatively thin cap layer of the quantum well was prepared during the growth. These results could facilitate high efficiency QW preparation, especially for green LEDs.

## 1. Introduction

When group III nitrides and their alloys are used for semiconductor device materials, they have a wide band gap range [1,2], and the emission wavelength covers the entire spectral range from infrared to ultraviolet [3,4]. Therefore, group III nitride semiconductors have been used in various colors of light-emitting diodes and laser diodes [5,6,7,8]. At present, InGaN/GaN multi-quantum wells are usually grown on inexpensive sapphire substrates through heteroepitaxy, whose performances are limited by two obstacles. First, due to the large lattice mismatch and thermal mismatch between sapphire and GaN, the GaN epitaxial material will have a large number of threading dislocations, and the dislocation density can reach as high as 10^8^–10^10^ cm^−2^ [9]. They act as non-radiative recombination centers of carriers, resulting in a decrease in the luminescence efficiency of the material [10,11]. Secondly, the InGaN material has spontaneous polarization and piezoelectric polarization. Polarization charges and polarization electric fields are generated at the quantum wells, which lead to a tilt in the energy band, in turn reducing the overlap ratio of electron-hole wave functions and the luminescence efficiency [12,13]. However, despite such high dislocation density and strong piezoelectric field, the luminescence efficiency of InGaN/GaN quantum wells is still very high, which can be attributed to the randomly distributed localized carrier confinement potential formed across the energy band [14,15,16]. The localized states localize the carriers so that the adverse effects of material defects and the quantum confined Stark effect (QCSE) on luminescence can be effectively suppressed [17]. Therefore, the localized states play an important positive role in the light-emitting diode (LED) luminescence of the active region of InGaN/GaN multiple quantum wells (MQWs). In this work, during the MOCVD growth of MQWs, a thin low-temperature GaN cap (LT-cap) layer was inserted after the growth of each InGaN well layer to protect the InGaN from decomposition during the later temperature rise, thereby improving the luminescence efficiency of the MQW. However, with the increase in the thickness of the low-temperature cap layer, severe indium segregation and a large number of defects may also be brought into the quantum well layers, which negatively affect the luminescence quality of the quantum wells [18]. Therefore, it is very important to analyze the influence of the thickness of the GaN cap layer on the localized state effect for the performance improvement of practical light-emitting devices. Since the content of InGaN In is higher in the QWs of green LEDs, it is more important to add a GaN cap layer to improve the quality of luminescence when the QWs are grown.

In this work, four InGaN/GaN MQW samples with the same green LED device structure were prepared using an MOCVD system, and the thicknesses of the well layer and cap layer were controlled only by changing the well layer growth time and LT-GaN cap layer growth time. The luminescence properties of the quantum wells were explored by analyzing the temperature-dependent photoluminescence spectra and micro-fluorescence imaging. We proposed a localized states homogeneity model to analyze the relationship between the cap layer thickness and the luminescence properties of quantum wells. It was found that properly reducing the thickness of the cap layer will not only reduce the point defect concentrations caused by the residual In atoms, but also improve the uniformity of the localized states, thus improving the luminescence characteristics of the quantum wells. Therefore, controlling the thickness of the cap layer is important to improve the luminescence performance of green quantum well LEDs.

## 2. Experiment

**Figure 1:**
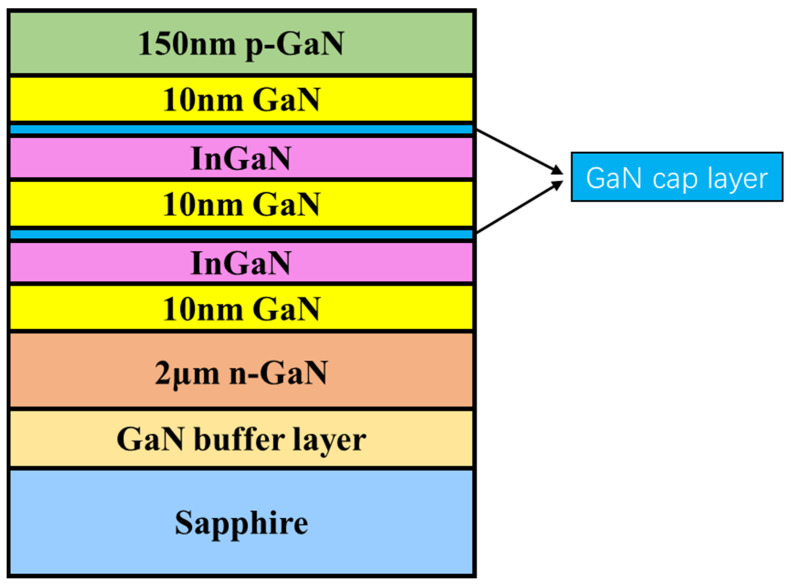
The schematic diagram of the epitaxial layer structure of the studied InGaN/GaN MQW samples, where the thickness of InGaN layer is between 2.5 nm to 3.0 nm, and the In composition of InGaN is between 11.7% to 13.6%. The parameter details are listed in Table 1.

InGaN/GaN MQW samples with different thicknesses of the well layer and cap layer were grown on c-plane sapphire substrates using an AIXTRON 3 × 2 inch close-coupled showerhead reactor MOCVD system. In the process of growth, trimethylindium (TMIn), trimethylgallium (TMGa), and ammonia (NH_3_) were used as the precursor gases for the In source, Ga source, and N source. The dopant of N-type GaN was silane (SiH_4_), and the dopant of P-type GaN was magnesium dicene (Cp_2_Mg). In addition, hydrogen H_2_ was used as the carrier gas for GaN growth, and nitrogen N_2_ was used as the carrier gas for InGaN growth. All samples were deposited layer by layer, from bottom to top, with a low-temperature GaN buffer layer on the sapphire substrate, a 2 µm thick n-GaN layer, a MQW active region formed by two InGaN/GaN quantum wells, and a 150 nm p-GaN layer. During the growth of each InGaN layer of the MQW active region, the TMIn flow rate was kept constant as 35 mL/min. After each InGaN well layer growth, a low-temperature GaN (LT-GaN) cap layer was grown at a temperature of 700 °C, and then the temperature was increased to perform an interface treatment. The GaN barrier layer was then grown at 830 °C. Except for the growth time duration of the well layer and the cap layer, all other growth conditions were the same for the 4 different samples, namely, A, B, C, and D, as shown in Table 1. It can be seen that from A to B and C the InGaN well layer growth time increased from 140 to 160 s, and then finally to 200 s, with D; from A and B to C and D, the cap layer growth time decreases from 120 s to 60 s. Figure 1 shows a schematic diagram of the epitaxial layer structure of these samples.

**Figure 2:**
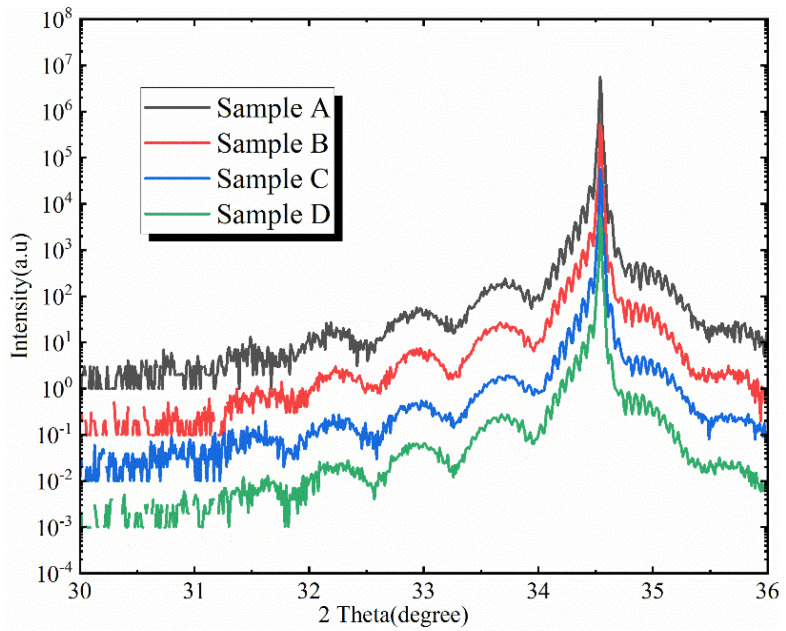
HRXRD ω–2θ scan curves for samples A, B, C, and D. From the ω–2θ curves of the InGaN/GaN quantum well structure, we can obtain the diffraction peaks of GaN and the periodic structure of the quantum wells. The In component in the quantum well layers and the thickness of the periodic structure are obtained by fitting and are shown in Table 1.

In order to study the changes in the localized states of the four samples, the samples were measured using the temperature-dependent photoluminescence (TDPL) method. The luminescence spectra in the temperature range of 30~300 K were measured. A 405 nm semiconductor laser was used as the excitation source, an FHR640 spectrometer from HORIBA and a photomultiplier were used for PL measurement, and the sample temperature was controlled using a CTI Cryogenics closed-loop refrigerator. The structural parameters of the MQWs were characterized via high resolution X-ray diffraction (HRXRD). The ω–2θ scan curves are shown in Figure 2. Finally, the micro-region fluorescence image of the samples were tested under the excitation of a 405 nm laser using a Nikon A1 confocal optical system.

## 3. Results and Discussion

**Figure 3:**
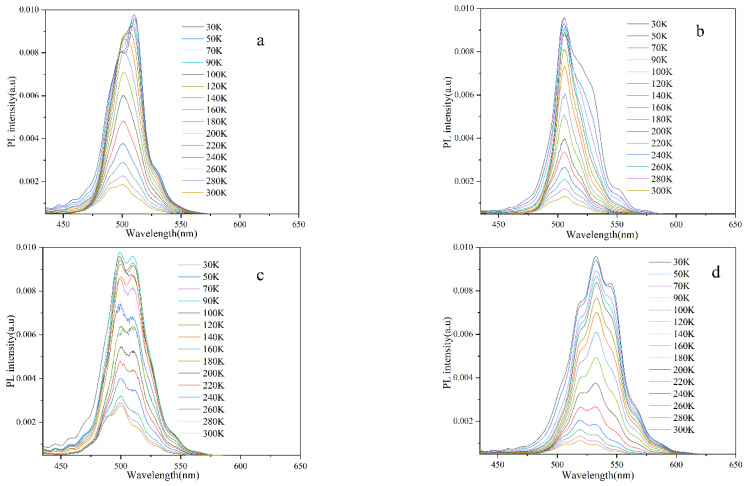
The photoluminescence spectra (**a**–**d**) of four samples, A, B, C, and D, measured at different temperatures.

As is well-known, there is a large dislocation density in InGaN/GaN MQWs grown on sapphire substrates, but their luminescence efficiency may be still very high due to the existence of more localized states existing in the energy gap. Therefore, the carrier localization effect of the InGaN/GaN quantum well has become one focus of researchers. Usually, the localized effect of carriers can be characterized and described by the “S”-shaped curve of peak energy variation with temperature; much information can be extracted from the test of photoluminescence spectra at variable temperature [19,20]. Figure 3 shows the overall photoluminescence spectra measured at different temperatures for each sample excited by a 405 nm laser. It can be seen that, except at very low temperature, the PL intensity generally decreases with the increase of temperature. However, the PL intensity of sample C is slightly enhanced from 50 K to 70 K. This abnormal increase may be due to the fact that carriers trapped by some non-radiative surface defects obtain thermal energy and migrate to a deep localized state with increasing temperature, which leads to an increase of the PL intensity from the MQW.

**Figure 4:**
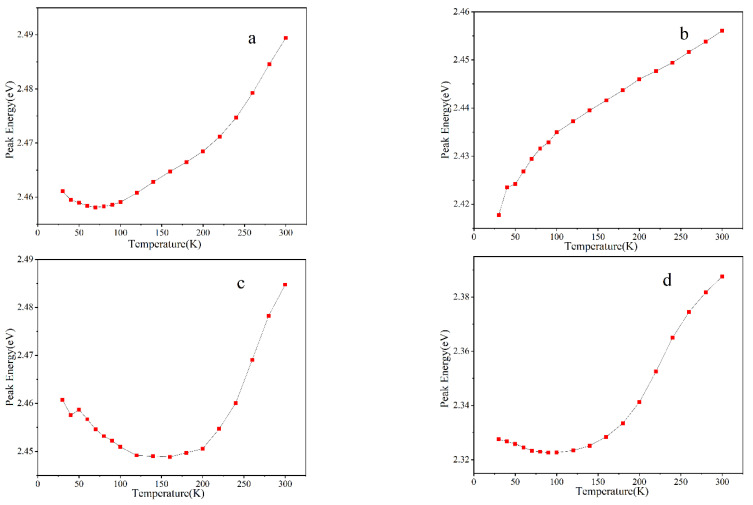
The temperature dependencies of PL peak luminescence energy (**a**–**d**), for four samples, A, B, C, and D.

Figure 4 shows the temperature-dependent luminescence peak energy of four samples excited by a 405 nm laser. The reason for choosing the 405 nm laser to excite PL is to avoid the influence of the luminescence from the GaN layers on the final result of QW luminescence.

It can be seen from Figure 4 that none of the four samples shows a typical “S”-shaped curve change in the temperature dependence of the luminescence peak energy [19]. With the increase in temperature, the position of the PL peak energy for sample A at first shows a weak red shift at low temperature, and then a rapid blue shift occurs after 70 K. The PL peak position of sample B has a blue shift with the increase of temperature, but the blue shift becomes relatively fast at 100 K, and it turns out to be relatively slow when the temperature exceeds 100 K. For sample C, the PL peak position exhibits a “V”-shaped change with increasing temperature. First, in the low-temperature range, with the increase of temperature, the peak position undergoes a red shift, and then a rapid blue shift occurs after 150 K. Compared to sample C, sample D exhibits a weak “V”-shaped change, with a slow red shift in the peak value before the temperature rises to 100 K, and then a faster blue shift. It is well-known [16] that the temperature-dependent shift of the PL peak is the result of competition effects between the thermal redistribution of carriers in the localized states and the effect of temperature-induced band gap shrinkage. At low temperature, the photo-generated carriers in the quantum wells do not have enough thermal energy to migrate to the localized states with the lowest energy. They are randomly distributed in all levels of localized states and undergo radiative recombination there. With the increase of temperature, the carriers obtain more thermal energy and have the ability to migrate to the localized state with the minimum energy and to undergo-radiative recombination there, resulting in a red shift in the PL peak. Then, with the further increase of temperature, the carriers obtain even more thermal energy and redistribute into the localized states with higher energy. If the thermal band gap shrinkage effect is comparatively weaker, then a blue shift of the luminescence peak will occur. As can be seen in Figure 4, none of the four studied samples shows a red-shift phenomenon appearing at high temperature, indicating that the carriers of the four samples are strongly redistributed in the localization states by heating, and the density of the localized states in these four samples is high. We can calculate the total amount of blue shift for each sample from low temperature to room temperature. It is about 0.032 eV for sample A, about 0.039 eV for sample B, about 0.037 eV for sample C, and about 0.065 eV for sample D. The results obtained from the four samples can be roughly analyzed as follows.

When the thickness of the cap layer is thick enough (as for samples A and B, where the cap layer growth time is 120 s instead of 60 s), as the growth time of the well layer increases, that is, from the case of sample A to the case of sample B, the red shift in the temperature dependence curve disappears, and the total blue shift increases. This means that at 30 K, the photo-excited carriers in sample B have already filled the lowest localized states and need more thermal energy to move away from one localized state to another, i.e., the carriers only jump from the lower energy level of localized states to the higher energy level, and there is no further carrier injection into the lower energy localized states. This indicates that the localized states of sample B are very numerous and more inhomogeneously distributed than in sample A. Then, when the cap layer growth time is reduced, that is, from sample B to sample C (the cap layer growth time of sample C is 60 s), in the low-temperature range, the red shift in the curve appears again, and the transition temperature at which the red shift–blue shift transition occurs is delayed until 150 K. From the above analysis, we can conclude that when the cap layer is relatively thick and the growth time of the well layer is increased, as it is from sample A to sample B, the localized state distribution in the wells will become non-uniform. When the growth time of the cap layer is shortened, that is, the thickness of the cap layer is reduced, which is observed from sample B to sample C, the uniformity of the localized states in the quantum wells will be improved.

According to previous reports, deep localized centers in quantum wells are mainly composed of In-rich InGaN clusters (some type of self-assembled In-rich quantum dots) formed by In segregation [21,22,23]. The In segregation is influenced by the growth of an LT GaN cap layer which directly covers the InGaN well layer. Under the cap layer, the floating In atoms at the InGaN well layer surface do not have enough time for desorption. Additionally, the In-rich InGaN cluster regions are formed. The above-mentioned In-rich quantum dot-like localization centers can serve as light-emitting centers, and the carriers confined to them cannot easily make contact with the dislocation defects to generate non-radiative recombination, which is considered to be an important reason for the high-efficiency luminescence of InGaN/GaN quantum well structures. On the other hand, it is known that there may also be a large number of defects in the In-rich InGaN cluster regions, especially if the size of the In-rich regions formed by a large amount of In segregation is too large and if their distribution is relatively non-uniform, which will inevitably lead to a possible decrease in the carrier recombination efficiency [24]. For sample A and sample B, the cap layer is thick, resulting in an increase in the number of residual In atoms on the surface. Therefore, as the growth time of the well layer increases, the In-rich clusters formed by In segregation will increase, which leads to the non-uniform distribution of localized states in sample B. However, when the thickness of the cap layer decreases, as is the case when going from sample B to sample C, the ability of the cap layer to protect the InGaN well layer is weakened, and the In atoms in the InGaN layer can migrate and desorb more easily in the subsequent heating process. The size of In-rich clusters formed by In segregation will be reduced, and the distribution of In-rich clusters will be more uniform, which will improve the uniformity of localized states in InGaN quantum wells. The decrease of the cap layer thickness also slightly affects the change in the well layer thickness. In order to obtain a more accurate conclusion, we extended the growth time of the well layer compared to sample C to obtain sample D. In comparison with sample B, sample D has the same well layer thickness, and it is found that a low-temperature red shift can be observed in the S-type temperature-dependent curve, which suggests that the localized state uniformity of sample D improved to be better than sample C. In summary, the uniformity of the localized state in the quantum wells was improved by appropriately reducing the thickness of the cap layer.

**Figure 5:**
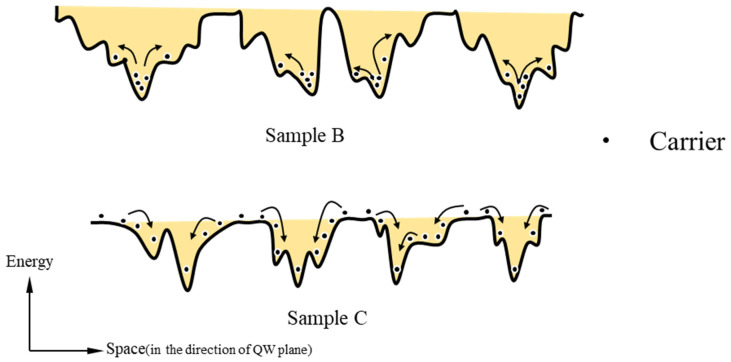
Schematic diagram of localized state distribution in samples B and C to illustrate how carriers are confined at low temperature. The arrows describe the main migration directions of carriers when the temperature is raised.

Figure 5 schematically shows the possible distribution of localized states in sample B and sample C at 30 K. The arrows represent the direction of carrier migration. It can be seen that the uniformity of the localized states in sample B is very poor, which is due to the thick cap layer, and the segregated In atoms are not easily desorbed away from the well layer and thus may migrate and form In-rich clusters that become too large in size. Meanwhile, there are a variety of localized states in the quantum wells. The carriers fill the low-energy localized state at low temperatures. When the temperature increases, the carriers in the low-energy localized state continue to migrate to the higher-energy localized state, resulting in a remaining blue shift of the PL peak as the temperature increases. When the thickness of the cap layer is reduced, the migration of In atoms becomes easier during the subsequent heating process, which improves the uniformity of the localized states, as observed for the localized states in sample C. In sample C, many carriers are not filled with the lowest localized states at low temperature, but when the temperature begins to rise, the carriers first migrate to the lowest localized states, and when the temperature rises further to a certain level, the carriers in the lowest localized states then leap to higher-energy states, at which time the position of the PL peak energy increases with increasing temperature and presents a V-shaped temperature dependence.

**Figure 6:**
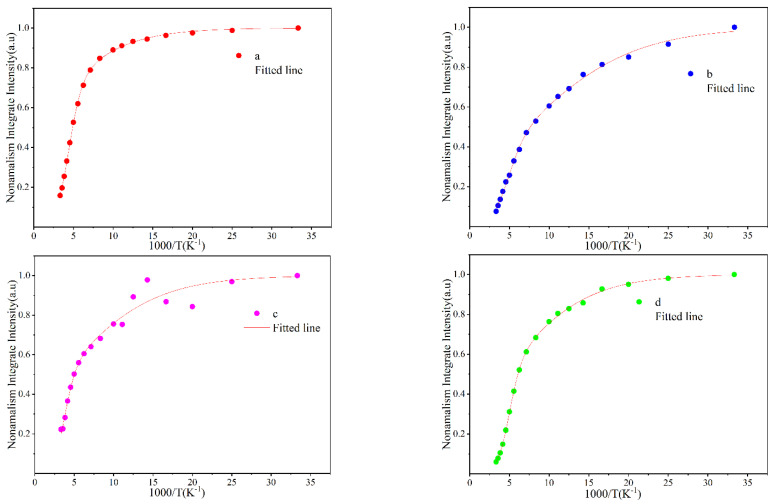
The temperature dependencies of the PL emission integrated intensity (**a**–**d**) of samples A, B, C, and D.

Figure 6 shows the four curves of the PL integrated intensity of the four samples as a function of temperature. The points in the figure are the experimentally measured data of integral intensity, and the red solid lines are the curves fitted using the Arrhenius formula [25,26]:

$$I\left(T\right)=\frac{1}{1+C_{1}\exp\left(-\frac{E_{a1}}{kT}\right)+C_{2}\exp\left(-\frac{E_{a2}}{kT}\right)}$$

where *Ea*_1_ and *Ea*_2_ are the activation energies of the nonradiative recombination centers, *C*_1_ and *C*_2_ are the constants related to the corresponding nonradiative recombination, and *k* is the Boltzmann constant.

It can be seen in Figure 6 that the temperature dependence curves of the integrated luminescence intensities of samples A, B, and D can be well-fitted using the Arrhenius formula, where the PL integrated intensity decreases with the increase of temperature. For sample C, however, the intensity first decreases, and then there is a small temperature region near the low temperature of 50 K where the intensity increases, which may be due to the fact that carriers trapped by some non-radiative surface defects obtain thermal energy and migrate to a deep localized state at increasing temperature, which leads to an increase of the luminescence integrated intensity. This explanation is also supported by the relatively small blue shift in the peak energy of sample C at 50 K in Figure 4. If we assume that the internal quantum efficiency (IQE) at the low temperature of 30 K is 100%, then the IQE of QWs at room temperature (RT) can be estimated by the ratio value of the PL integrated intensity at room temperature to the integrated intensity at 30 K. The RT IQE for samples A, B, C, and D is estimated to be 15.8%, 7.6%, 22.3%, and 6.0%, respectively. It can be seen that properly shortening the growth time of the GaN cap layer can remarkably improve the RT internal quantum efficiency of the material and the uniformity of the localized states. For example, the growth time of the well layer of sample C is 160 s, but the growth time of the cap layer is only 60 s, the latter being relatively thin. The RT IQE of sample C is the highest among the four samples. As shown in Figure 3, an abnormal increase in the luminescence intensity is observed when the temperature of sample C increases from 50 K to 70 K. This phenomenon might be related to the fact that the luminescence centers induced by the localized states in sample C are more uniformly distributed, and fewer defects are contained, which may help to make the PL intensity enhancement with increasing temperature from 50 K to 70 K possible.

**Figure 7:**
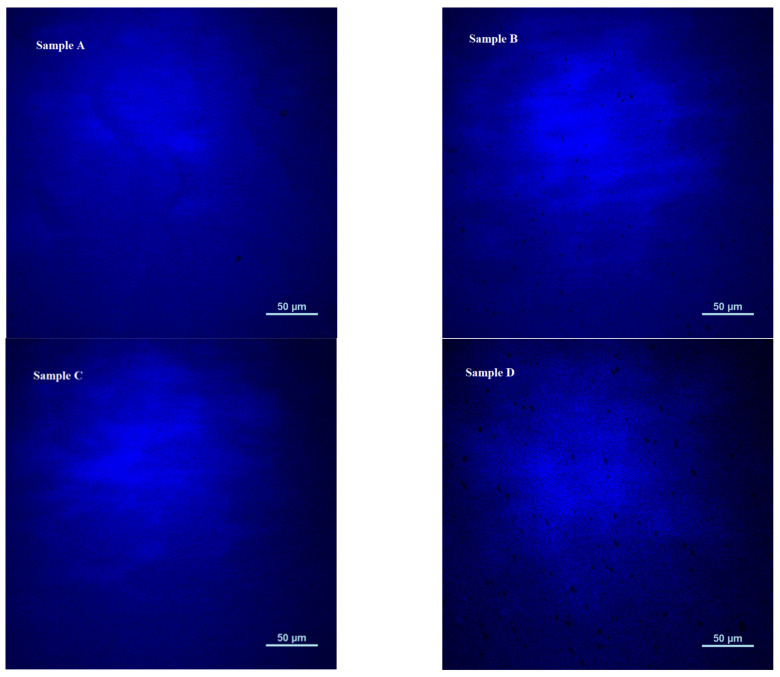
Microscopic fluorescence images of samples A, B, C, and D.

In addition, the micro-region fluorescence images of samples A, B, C, and D were measured via confocal microscopy and the results are shown in Figure 7. The black spots (dark areas in the micro-images) represent the non-luminous areas in the samples. As the growth time of the well layer increases, that is, from sample A to sample B, the dark spots in the micro-region fluorescence image increase. Subsequently, by reducing the growth time of the cap layer, from sample B to both sample C and sample D, it can be found that the number of black spots in sample C is significantly reduced, and the number of black spots in sample D is reduced but their size is increased, which may be due to the growth of the well layer with a longer time. Partial relaxation occurs, and the aggregation of residual In atoms is more serious, resulting in the introduction of additional defects. Therefore, this phenomenon also verifies our above-mentioned analysis from another side: using a thinner cap layer can improve the uniformity of the quantum well localized states and improve the luminescence performance.

## 4. Conclusions

We have studied the effects of the thickness of the LT-GaN cap layer grown on the top of each InGaN quantum well layer on the distribution of the localized states and luminescence properties of InGaN/GaN quantum wells grown using an MOCVD system. The effect of the cap layer thickness (in correlation with the well layer thickness) on the luminescence properties of the quantum wells was studied by comparing and analyzing their TDPL spectra and micro-area fluorescence images. Through the temperature-dependent (TD) PL spectra, the localized state model is proposed to show that an appropriate reduction of the thickness of LT-cap layers and well layers can not only improve the uniformity of the localized states in the quantum wells, but also reduce the defects formed by the segregation of residual In atoms. The luminescence performance of QWs will be significantly improved, especially for green light LEDs, whose QWs have a higher In content.

## Figures and Tables

**Table 1:** Growth time and thickness of InGaN/GaN multiple quantum well layers where a GaN cap layer was grown on each InGaN well layer. The cap layer thickness was estimated from the growth time and speed. The well layer thickness and In composition were measured by XRD.

Sample	Cap Layer Growth Time	Cap Layer Thickness (ca.)	Well Layer Growth Time	Well Layer Thickness	In Content of InGaN
A	120 s	1.00 nm	140 s	2.83 nm	11.70%
B	120 s	1.00 nm	160 s	2.93 nm	13.20%
C	60 s	0.50 nm	160 s	2.43 nm	13.60%
D	60 s	0.50 nm	200 s	2.93 nm	13.50%
